# Quantitative Proteomic Analysis of Mouse Sciatic Nerve Reveals Post-injury Upregulation of ADP-Dependent Glucokinase Promoting Macrophage Phagocytosis

**DOI:** 10.3389/fnmol.2021.777621

**Published:** 2021-11-12

**Authors:** Kai Zhang, Qingyao Wang, Yiyao Liang, Yu Yan, Haiqiong Wang, Xu Cao, Bing Shan, Yaoyang Zhang, Ang Li, Yanshan Fang

**Affiliations:** ^1^Interdisciplinary Research Center on Biology and Chemistry, Shanghai Institute of Organic Chemistry, Chinese Academy of Sciences, Shanghai, China; ^2^University of Chinese Academy of Sciences, Beijing, China; ^3^Guangdong Key Laboratory of Non-human Primate Research, Guangdong-Hong Kong-Macau Institute of CNS Regeneration, Jinan University, Guangzhou, China; ^4^Bioland Laboratory (Guangzhou Regenerative Medicine and Health Guangdong Laboratory), Guangzhou, China; ^5^Key Laboratory of CNS Regeneration (Jinan University), Ministry of Education, Guangzhou, China; ^6^Department of Neurology, Guangdong Neuroscience Institute, Guangdong Provincial People’s Hospital, Guangdong Academy of Medical Sciences, Guangzhou, China

**Keywords:** peripheral nerve injury, axon degeneration, macrophage, neuroimmune, proteomics

## Abstract

Nerve injury induces profound and complex changes at molecular and cellular levels, leading to axonal self-destruction as well as immune and inflammatory responses that may further promote neurodegeneration. To better understand how neural injury changes the proteome within the injured nerve, we set up a mouse model of sciatic nerve injury (SNI) and conducted an unbiased, quantitative proteomic study followed by biochemical assays to confirm some of the changed proteins. Among them, the protein levels of ADP-dependent glucokinase (ADPGK) were significantly increased in the injured sciatic nerve. Further examination indicated that ADPGK was specifically expressed and upregulated in macrophages but not neurons or Schwann cells upon injury. Furthermore, culturing immortalized bone marrow-derived macrophages (iBMDMs) *in vitro* with the conditioned media from transected axons of mouse dorsal root ganglion (DRG) neurons induced ADPGK upregulation in iBMDMs, suggesting that injured axons could promote ADPGK expression in macrophages non-cell autonomously. Finally, we showed that overexpression of ADPGK *per se* did not activate macrophages but promoted the phagocytotic activity of lipopolysaccharides (LPS)-treated macrophages. Together, this proteomic analysis reveals interesting changes of many proteins within the injured nerve and our data identify ADPGK as an important *in vivo* booster of injury-induced macrophage phagocytosis.

## Introduction

Injury can cause disconnection and destruction of nerves, leading to partial or total loss of motor, sensory and autonomic functions of the nervous system (Caillaud et al., 2019; Vijayavenkataraman, 2020). Upon neural injury, a cascade of complex biological reactions occur, including dendritic retraction, axonal degeneration, chromatolysis, decomposition of the myelin sheath, infiltration of immune cells, and removal of axon and myelin debris by phagocytosis, followed by regenerative events in the injured nerve (Lee and Wolfe, 2000; Navarro et al., 2007; Zhang et al., 2021). In humans, although the peripheral nervous system is capable of regeneration, a complete functional recovery of the injured peripheral nerve often takes months to years, and in some cases is never achieved (Sulaiman and Gordon, 2013). Hence, revealing and understanding the molecular changes in peripheral nerve injury (PNI) become a prerequisite for translational study and the development of novel therapeutics to treat degeneration and promote regeneration in PNI.

Recent biotechnology innovations and improvements have significantly advanced our understanding of biology and human diseases including PNI. For example, microarray and RNA-sequencing have been used to unmask molecular alterations in the injured nerve at the transcriptomic level, some of which have suggested a key role of macrophages in modulating neuroinflammation and subsequently affecting the prognosis (Vega-Avelaira et al., 2009; Ydens et al., 2020; Niehaus et al., 2021). Indeed, the resident macrophages (2–9% of total cells in peripheral nerves) secrete proinflammatory cytokines and chemokines that promote neuroinflammation in the acute phase of nerve injury (Mueller et al., 2001; Schuh et al., 2014; DeFrancesco-Lisowitz et al., 2015). In the later phases, macrophages not only participate in the phagocytosis and clearance of axon and myelin debris, but may also stimulate neuroregeneration by polarization to the anti-inflammatory M2 state (Chen et al., 2015).

Alterations of gene expression at the RNA level, although important, may not faithfully or timely reflect the changes at the protein level (Vogel and Marcotte, 2012). As such, biologists including neuroscientists have been embracing the mass spectrometry (MS)-based, high-throughput proteomics in recent years (Craft et al., 2013). In particular, tandem mass tag (TMT), which facilitates sample multiplexing and accurate quantification of peptides and proteins in MS-based proteomics, allows multiple samples to be analyzed simultaneously and thus has caught the attention of researchers in the field of PNI. However, the existing proteomic studies of PNI often focused on only a specific and pre-defined aspect, such as the proteome of axonal transport (Michaelevski et al., 2010), Schwann cells (Shi et al., 2018) and denervated skeletal muscles (Sun et al., 2012, 2014). In this study, we conducted an unbiased, quantitative proteomic analysis of PNI using a mouse SNI model, which identifies ADPGK as a potential booster of macrophage phagocytosis in nerve injury.

## Materials and Methods

### Animal Care and Surgery

All practices on mice in this study were performed in compliance with the institutional guidelines on the scientific use of living animals at Interdisciplinary Research Center on Biology and Chemistry, the Chinese Academy of Sciences, as well as “Principles of Laboratory Animal Care” by National Institute of Health, United States (No. 86-23). Animal distress and conditions requiring euthanasia were addressed and the number of animals used was minimized. Eight- to twelve-week-old C57BL/6J male mice purchased from the Charles River Laboratories (Beijing) were housed at 24°C and 60% humidity on a 12 h:12 h light/dark cycle and allowed for free access to water and food. Mouse SNI was performed as previously described (Bauder and Ferguson, 2012). In brief, a mouse was anesthetized with 8% chloral hydrate, and its left sciatic nerve was exposed through an incision on the lateral aspect of the mid-thigh of the left hind limb. The crush lesion was conducted using a pair of hemostatic forceps for 10 s. The right sciatic nerve of the same mouse received sham-surgery and was used as an internal control.

### Antibodies

The following primary antibodies were used in this study: anti-ADPGK (15639-1-AP), anti-RAC2 (10735-1-AP), anti-CA1 (13198-2-AP) and anti-MPO (22225-1-AP) from Proteintech; anti-MBP (sc-271524), anti-F4/80 (C-7) (sc-377009) and anti-c-Myc (9E10) (sc-40) from Santa Cruz; anti-NFH (Abcam, ab4680), anti-Tuj1 (Sigma-Aldrich, T2200) and anti-Actin (Cell Signaling Technology, 3700). HRP-conjugated secondary antibodies: anti-mouse (Sigma-Aldrich, A4416), anti-rabbit (Sigma-Aldrich, A9169) and anti-chicken (Abcam, ab6877). Fluorescent secondary antibodies (all from Life Technologies): anti-rabbit Alexa Fluor 633 (A21071), anti-mouse Alexa Fluor 488 (A32723), anti-rat Alexa Fluor 488 (A11006), anti-rabbit Alexa Fluor 568 (A11011) and anti-chicken Alexa Fluor 568 (A11041).

### Mass Spectrometry Sample Preparation and Tandem Mass Tag Labeling

The mice were euthanized by overdose of the anesthesia (8% chloral hydrate) and immediately perfused with ice-cold PBS (Sangon, SD8117). The distal sciatic nerves with or without crushing were micro-dissected from the euthanized mice at indicated time points after injury, and snap frozen to −80 °C. The tissues were homogenized using Tissue Extraction Reagent I (Thermo Fisher Scientific, FNN0071) with 0.1% sodium dodecyl sulfate (SDS). The protein concentrations were measured using the Pierce™ BCA Protein Assay Kit (Thermo Fisher Scientific, 23225) and adjusted to 1 μg/μL with 100 mM triethylammonium bicarbonate (TEAB).

The following reagents were added to 100 μL of the adjusted nerve lysates sequentially: 1 μL of 1 M Tris 2-carboxyethyl phosphine (55°C, 1 h), 3.75 μL of 500 mM iodoacetamide (25°C, 30 min) and 600 μL of pre-chilled (−20°C) acetone, and allowed to precipitate overnight. On the next day, samples were centrifuged at 8,000 × *g* for 10 min at 4°C. Pellets were collected and resuspended in 100 μL of 100 mM TEAB with vortexing and sonication, followed by trypsin digestion (25 μg/mL) (Promega, V5113) at 37°C overnight.

TMT labeling was conducted using the TMT10plex™ Isobaric Label Reagent Set (Thermo Fisher Scientific, 90110) according to the manufacturer’s instruction. The TMT labeling reaction was terminated with addition of 8 μL of 5% hydroxylamine into each sample and incubation at room temperature for 15 min. Four samples of different conditions were pooled into one vial, air-dried, fractionated using high pH reverse phase HPLC, and then re-dissolved with 50 μL of 0.1% formic acid (FA) for the subsequent MS analysis.

### Liquid Chromatography-Tandem Mass Spectrometry (LC-MS/MS) Analysis

The TMT-labeled peptides were analyzed using an online system consisting of an EASY-nLC 1200 and a Q-Exactive HF mass spectrometer under the control of Xcalibur Software (Thermo Fisher Scientific). Briefly, 1 μg of the resuspended peptides from each sample fraction was automatically loaded and analyzed by a 15-cm capillary analytical column that was packed in-house with 1.9 μm of C_18_ ReproSil particles (Dr. Maisch GmbH). The mobile phase A buffer included 2% acetonitrile and 0.1% FA, while the phase B buffer comprised 98% acetonitrile and 0.1% FA, with a flow rate of 300 nl/min. The 120-min gradient was carried out as the following: 5–10% B for 2 min, 10–20% B for 85 min, 20–35% B for 22 min, 35–60% B for 4 min, 60–90% B for 2 min and 90% B for 5 min.

The LC-MS/MS data were collected in a data-dependent acquisition mode. The full mass scans were acquired at a resolution of 60,000 and the scan range was 100–1,500 m/z. The top 20 precursor ions were selected for HCD fragmentation with a normalized collision energy of 37% and a dynamic exclusion time of 30 sec. The MS/MS scans were similarly acquired at a resolution of 30,000. The AGC targets for the full and MS/MS scans were set to 1 × 10^6^ and 2 × 10^5^, respectively. The spray voltage of the ESI ion source was 2.3 kV and the temperature of the ion transfer capillary was 350°C.

### LC-MS/MS Data Processing

Protein database searching of all the raw LC-MS/MS data was performed with Thermo Proteome Discoverer 1.4 (Thermo Fisher Scientific) using the SequestHT search engine against the complete *Mus musculus* sequence database including all UniProt entries (downloaded on Jan 10, 2017). The spectral selection and most other parameters were set at the default settings. Trypsin was specified as the protease. Mass tolerance for precursor and fragment ions was 10 ppm and 0.6 Da, respectively. Fixed modifications were set as TMT at peptide N-termini and lysine residues, and carbamidomethyl at cysteine residues. Methionine oxidation was set as a variable modification. Posterior error probabilities were calculated and peptide spectrum matches were filtered using Percolator. False discovery rate was estimated with a *q*-value < 0.05. For reporter ion quantification in a consensus workflow, the normalized intensity of unique and razor peptides was extracted for subsequent processing and statistical analysis.

The difference in protein levels was determined with *t*-tests using R (Version 4.0.5), and Package ggplot2 (Version 3.3.3) and Package pheatmap (Version 1.0.12) were used in the data profiling. Gene ontology (GO) enrichments of the differentially expressed proteins were analyzed using DAVID Bioinformatics Resources 6.8^1^ (Huang da et al., 2009a,b) and GO category^2^ (Ashburner et al., 2000).

### Plasmids

To generate the pCDH-CMV-*myc-ADPGK* plasmid, the DNA fragment encoding mouse *ADPGK* was amplified with the cDNA from the mouse lung by PCR, using the following primers:

*ADPGK*-F*:*

5′-ATGGCCATGGAGGCCGAATTCATGGCGCTGTGGCGCGGCTC-3′

*ADPGK*-R*:*

5′-CATGTCTGGATCCCCGCGGCCGCCTAATCAGGGCGTGCTTCTG-3′

The PCR product was sub-cloned into a pCMV-*myc* vector using the EcoRI/NotI sites. The DNA fragments of *myc-ADPGK* were amplified from the above plasmid and sub-cloned into a pCDH-CMV-MCS-EF1-Puro vector at the EcoRI/BamHI sites. The primers used are:

*myc-ADPGK*-F:

5′-AGATTCTAGAGCTAGCGAATTCGCCACCATGGCATCAATGCAGAAGCT-3′

*myc-ADPGK*-R:

5′-ATCCTTCGCGGCCGCGGATCCCTAATCAGGGCGTGCTTCTG-3′

### Cell Culture, Lentivirus Production, and Immortalized Bone Marrow-Derived Macrophage Infection

iBMDMs and 293T cells were cultured in Dulbecco’s Modified Eagle Medium (DMEM; Sigma-Aldrich, D0819) containing 10% fetal bovine serum (FBS) and 1% penicillin-streptomycin (Invitrogen, 10378016). To generate lentivirus for infecting iBMDMs, 293T cells at a confluency of 80–90% were co-transfected with pCDH-CMV-*myc*, or pCDH-CMV-*myc-ADPGK* together with psPAX2 and pMD2.G with a ratio of 4:3:2 in DMEM using PolyJet™ (SignaGen Laboratories, SL100688). The supernatants of the cell culture were harvested at 48 h after transfection and passed through a 0.45-μm polyvinylidene fluoride (PVDF) filter. Fresh supernatants containing viral particles were stored at 4°C until use. For lentiviral infection, the viral particles were mixed with polybrene and added to iBMDMs for 24 h.

### Phagocytosis Assay of Immortalized Bone Marrow-Derived Macrophages

The 1-μm, IgG-coated FluoSpheres™ Sulfate Microspheres (Invitrogen, F8852) were prepared according to the manufacturer’s instruction and stored at 4°C until use. To assess the phagocytotic capacity, the beads were added to iBMDMs for 2 h and the ones engulfed by the iBMDMs were imaged and counted under a Leica TCS SP8 confocal microscope. For experiments involving LPS induction, iBMDMs were pretreated with LPS (100 ng/mL for 24 h) prior to exposure to the beads.

### *In vitro* Mouse Dorsal Root Ganglion Cultures, Axotomy, and Conditioned Medium

Mouse DRG were dissected from C57BL/6J mice at embryonic Days 13–15 as previously described (Wang and Marquardt, 2012). Individual DRG explants were cultured in Transwell dishes (Corning, 35302) in serum-free Neurobasal medium (Invitrogen, 21103049) supplemented with 2% B27 (Invitrogen, 17504044), GlutaMAX (Invitrogen, 35050061) and 1% penicillin-streptomycin. On 7 days *in vitro* (DIV), DRG axons were severed by scraping the upper side of the Transwell insert containing somas. The remaining axons on the lower side of the insert were fixed for immunostaining. The axon media collected at 0, 6, 12, and 24 h after axotomy were centrifuged at 1,000 × *g* for 3 min and passed through a 0.45-μm PVDF filter. The axon-derived conditioned medium was added at a 1:1 ratio to the iBMDM culture medium and incubated for 6 h before the iBMDMs were lysed for western blotting analysis.

### Protein Extraction and Western Blotting

The mouse sciatic nerve was lysed in the ice-cold Tissue Extraction Reagent I containing protease inhibitor cocktail (Roche, 5892791001). iBMDMs were lysed in RIPA (50 mM Tris pH 8.0, 150 mM NaCl, 1% NP-40, 5 mM EDTA, 0.5% sodium deoxycholate, 0.1% SDS). All protein samples were boiled at 95°C for 5 min before separation by electrophoresis in a 10% Bis-Tris SDS-PAGE gel (Invitrogen, NP0303BOX) and probed with the specific antibodies listed above. Actin was used as a loading control for normalization. Detection was performed using the High-sig ECL Western Blotting Substrate (Tanon, 180-5001), and images were captured with an Amersham Imager 600 (GE Healthcare). The intensity of lanes was measured using ImageJ (National Institute of Health, United States). The contrast and brightness were optimized equally in Adobe Photoshop.

### Immunostaining and Confocal Microscopy

For immunohistochemistry, mice were deeply anesthetized by intraperitoneal injection of 8% chloral hydrate at the indicated time points after injury, and sequentially perfused with ice-cold PBS and 4% paraformaldehyde (PFA). For wholemount staining, the mouse sciatic nerve was fixed in 4% PFA at 4°C for 5 h. After removal of the epineurium, the nerve tissues were blocked with 10% goat serum in 0.1% Triton X-100 (Sigma-Aldrich, X100) in PBS (PBST) at 4°C overnight. For cryosection, the mouse sciatic nerve was fixed in 4% PFA at 4°C for 12 h, dehydrated with 30% sucrose, embedded in OCT compound (Tissue-Plus) and sectioned at a thickness of 10 μm with Leica CM1950 cryostat. The cryosections were washed with PBS and permeabilized with 0.3% Triton X-100. The samples were further incubated with Antigen Retrieval Solution (Solarbio, C1035) for 5 min and 10% goat serum in PBST for 30 min at room temperature. For immunocytochemistry, iBMDMs or DRG axons were fixed in 4% PFA for 15 min, permeabilized with 0.3% Triton X-100, and blocked with 3% normal goat serum in PBST.

All tissue and cell samples were incubated with primary antibodies at 4°C overnight and secondary antibodies at room temperature for 2 h. Slides were mounted using VECTASHIELD Antifade Mounting Medium with DAPI (Vector Laboratories, H-1200-10) and imaged with Leica TCS SP8 confocal microscopy system.

### Quantifications and Statistical Analyses

Statistical significance is determined by Student’s *t*-test with equal variances at *p < 0.05, **p < 0.01, and ***p < 0.001 as indicated. Error bars represent the standard error of the mean (SEM).

## Results and Discussion

### A Mouse Model of Peripheral Nerve Injury and the Setup for the Proteomic Analysis

**FIGURE 1:**
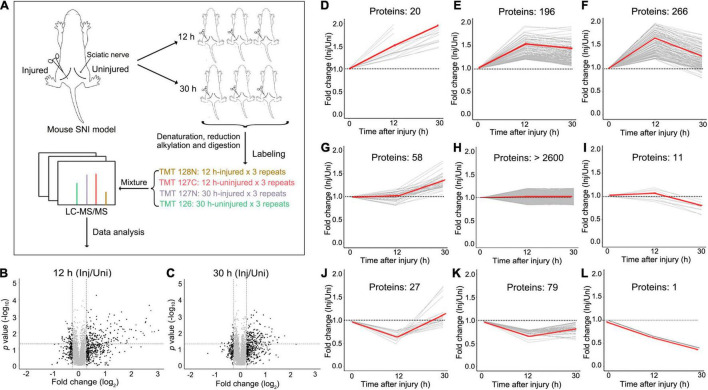
The experimental design and overview of the data of the quantitative proteomic analysis of mouse SNI. **(A)** The model of mouse SNI and the experimental design for the LC-MS/MS analysis. The left sciatic nerve of the mouse was injured, and the right sciatic nerve of the same mouse received sham surgery and was used as an internal control. Both injured and uninjured nerve samples were collected at 12 h or 30 h after injury for the subsequent LC-MS/MS analysis. For each time point, 3 mice were examined. **(B,C)** The scatter plots showing the proteins detected by the LC-MS/MS analysis at 12 h **(B)** or 30 h **(C)** after SNI. The fold change (injured/uninjured) and the statistical *p*-value of each protein detected are shown. Inj, injured; Uni, uninjured. **(D–L)** Different patterns of protein changes in the injured mouse sciatic nerve over time. 20 proteins are continuously increased at 12 and 30 h **(D)**; 196 proteins are increased at 12 h and then unchanged at 30 h **(E)**; 266 proteins are increased at 12 h and then decreased at 30 h **(F)**; 58 proteins are unchanged at 12 h but then increased at 30 h **(G)**; over 2600 proteins are unchanged at either time point **(H)**; 11 proteins are unchanged at 12 h but then decreased at 30 h **(I)**; 27 proteins are decreased first at 12 h and then increased at 30 h **(J)**; 79 proteins are decreased at 12 h and then unchanged at 30 h **(K)**; 1 protein kept decreasing at both 12 and 30 h after injury **(L)**. Of note, a theoretic value of 1 is set for the 0 h time point (injured/uninjured at 0 h), which serves as a baseline for the comparison of the proteomic changes at 12 and 30 h.

To gain an unbiased view of the changes of the proteome in the peripheral nerves in response to injury, we set up a mouse model of SNI by a crush injury (see section “Materials and Methods”). We first examined the axonal integrity of injured sciatic nerve at different time points (0, 12, 30, 40, and 48 h) by immunostaining for neurofilament heavy chain (NFH). The morphology of the sciatic nerve was normal and the axons were intact at 0 and 12 h after injury. At 30 h, most axons were intact while some showed minor roughness, and marked axonal disintegration and beading were detected at 40 h. By 48 h, substantial axonal fragmentation and degeneration were observed (Supplementary Figure 1). In the subsequent proteomic experiments in this study, we chose the time points of 12 h for the acute phase and 30 h for the pre-degeneration phase (Figure 1A). We did not examine the later time points because of the concern that once the injured nerve started to degenerate, the axonal self-destruction and the breakup of the axonal membrane might complicate the interpretation of the proteomic changes.

Unlike other studies of nerve injury, we did not set up a separate control group of mice receiving sham surgery. Instead, the experimental design of this study was to injure the left sciatic nerve and always perform the sham surgery on the right sciatic nerve of the same mouse (Figure 1A). This allowed us to use the uninjured right sciatic nerve as an internal control to normalize for the variation of the proteomes across different animals. And for each time point, the replicates of at least three mice were examined. Paired *t*-test was used to determine the statistical significance between the injured and uninjured nerves at each time point. Thus, in this study, the proteomic data were shown as relative fold changes (injured/uninjured) at 12 and 30 h; the group of 0 h (which would be injured/uninjured at 0 h after injury) was not applicable.

### Proteomic Profiling of Injured Mouse Sciatic Nerve

With TMT labeling and the LC-MS/MS analysis (see section “Materials and Methods”), we detected a total of 3289 proteins with assigned peptides from all samples examined (Figures 1B,C). Among them, the levels of 658 proteins were changed in the injured mouse sciatic nerve at either 12 or 30 h (fold change > 1.2 or < 0.83). Interestingly, the number of upregulated proteins was remarkably greater than that of downregulated proteins, in line with previous studies reporting that translation occurred locally within axons (Cajigas et al., 2012; Terenzio et al., 2018; Hafner et al., 2019) and protein synthesis was increased in injured peripheral nerves (Melemedjian et al., 2013). The changed proteins were further divided into 9 classes according to the patterns of their changes (Figures 1D–L), indicating that injury induced profound and differential changes of the nerve proteome.

**FIGURE 2:**
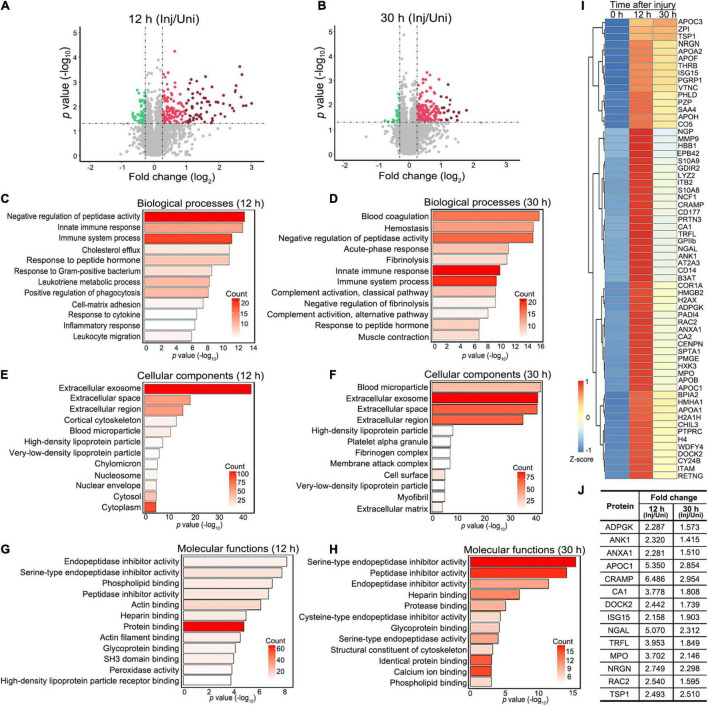
Proteomic profiling and functional annotation of injured mouse sciatic nerve. **(A,B)** The volcano plots highlighting the proteins that are significantly and consistently changed at 12 h **(A)** or 30 h **(B)** after injury in the mouse sciatic nerve. Fold change (injured/uninjured) > 1.2 or < 0.83 and *p*-value < 0.05. Green dots, downregulated proteins. Red dots, upregulated proteins; dark red dots, highly upregulated proteins (fold change > 2 and *p-*value < 0.05). **(C–H)** Gene ontology (GO) term analyses of significantly changed proteins at 12 **(C,E,F)** and 30 h **(D,F,H)** regarding their involvement in biological processes **(C,D)**, cellular components **(E,F)** and molecular functions **(G,H)**. The top 12 terms (*p*-value) and the counts of changed proteins in each term indicated by the color ramp are shown. **(I)** The heatmap showing the clustering of 63 proteins that are highly increased at 12 h or 30 h after injury (the dark red dots in A and B). Fold change (injured/uninjured) > 2 and *p*-value < 0.05. **(J)** The table showing 14 proteins from **(I)** that are associated with known or potential functions in the nervous system. The average fold changes of each protein (injured/uninjured) at 12 and 30 h after injury are shown. Inj, injured; Uni, uninjured.

Specifically, 192 proteins were significantly increased (Supplementary Table 1) and 55 decreased (Supplementary Table 2) at 12 h (Figure 2A), while 167 proteins were significantly elevated (Supplementary Table 3) and 28 reduced (Supplementary Table 4) at 30 h (Figure 2B) after injury in the mouse sciatic nerve (fold change of injured/uninjured > 1.2 or < 0.83 and *p* value < 0.05). Gene ontology (GO) analyses of the changed proteins revealed that they were most enriched in immune and inflammatory responses along with other related biological processes at both 12 and 30 h after injury (FDR < 0.05) (Figures 2C,D). At the cellular level, the increased proteins were most enriched in the term of extracellular exosome along with other cellular components at both the 12 and 30 h after injury (Figures 2E,F), supporting active secretion and signaling transduction across different cells in the injured nerve. At the molecular level, protein binding was the most associated functional term at 12 h after injury (Figure 2G); at 30 h after injury, proteins were highly enriched in the GO terms of serine-type endopeptidase and other peptidase inhibitor activity (Figure 2H), which suggested a profound reorganization of the proteolysis system prior to the injury-induced axonal destruction and other degenerative processes such as demyelination (Zhang et al., 2021).

### ADP-Dependent Glucokinase Is Increased in the Injured Sciatic Nerve and Co-localized With Macrophages

Next, we sought to validate the changes of the proteins detected in the proteomic analysis and to further explore the physiological relevance of their changes in nerve injury. To narrow down the candidates to a testable list, we applied more stringent selection criteria – fold change > 2 and *p*-value < 0.05 at either time point, by which 63 proteins were defined as highly changed proteins (Figure 2I). By data mining^3^ and literature research, we found that at least 14 of the above proteins were previously reported or associated with functions in the nervous system (Figure 2J).

In a pilot experiment, we tested four of these proteins whose antibodies were commercially available, including ADPGK, CA1, RAC2, and MPO. By western blotting, we confirmed that the protein levels of ADPGK (Supplementary Figures 2A,B), CA1 (Supplementary Figures 2C,D) and RAC2 (Supplementary Figures 2E,F), but not MPO (Supplementary Figures 2G,H) were significantly increased in the mouse sciatic nerve at 12 h after injury. Since the change of ADPGK levels was robust and showed the highest statistical significance (Supplementary Figures 2A,B) among the four candidates tested in the pilot experiment, we focused on ADPGK in the rest of this study and the others were to be investigated in separate studies in the future.

**FIGURE 3:**
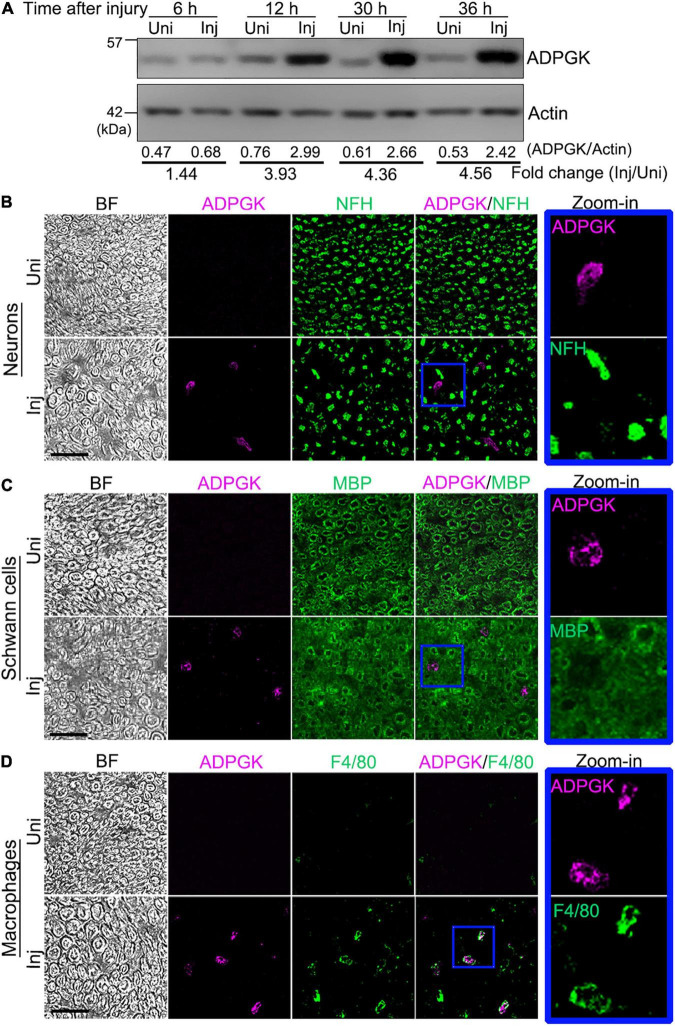
ADPGK is increased in the injured sciatic nerve and co-localized with macrophages. **(A)** Western blot analysis of ADPGK in the mouse sciatic nerve at indicated time points after injury. The relative protein levels of ADPGK (normalized to Actin) and the fold change (injured/uninjured) are shown. Also see Supplementary Figures 2A,B. **(B–D)** Representative images of the immunohistochemistry analysis of the cross sections of the mouse sciatic nerve co-immunostained for ADPGK and the neuro-axonal marker NFH **(B)**, Schwann cell marker MBP **(C)** or macrophage marker F4/80 **(D)** at 12 h after injury. Higher magnification images (zoom-in) of the individual channels of the blue-boxed areas are shown by the side. BF, bright field. Uni, uninjured; Inj, injured. Scale bars: 25 μm.

ADPGK plays an important role in glycolysis by catalyzing the ADP-dependent phosphorylation of glucose to glucose-6-phosphate. It was speculated that ADPGK might decrease the priming cost for the phosphorylation of glucose and save ATP in diseased conditions such as ischemia and hypoxia (Ronimus and Morgan, 2004). Indeed, we found that the protein levels of ADPGK were increased in the mouse sciatic nerve as early as 12 h and kept raising at 30 and 36 h after injury (Figure 3A). Furthermore, we did the immunohistochemistry assays of the mouse sciatic nerve before and 12 h after injury (Figures 3B–D). The results not only confirmed the injury-induced upregulation of ADPGK but also revealed that the induced ADPGK was specifically co-localized with the marker of macrophages (F4/80) (Figure 3D) but not neurons (NFH) (Figure 3B) or Schwann cells (myelin basic protein, MBP) (Figure 3C) in the injured mouse sciatic nerve.

### Injured Axons Induce ADP-Dependent Glucokinase Expression in Macrophages in a Non-cell Autonomous Manner

Since neural injury could recruit macrophages to the damaged nerve from the peripheral circulation (Fissel and Farah, 2020), it was not clear whether the increase of ADPGK in the injured sciatic nerve merely reflected the injury-induced infiltration of macrophages or there was indeed an upregulation of ADPGK in macrophages in response to neural injury.

**FIGURE 4:**
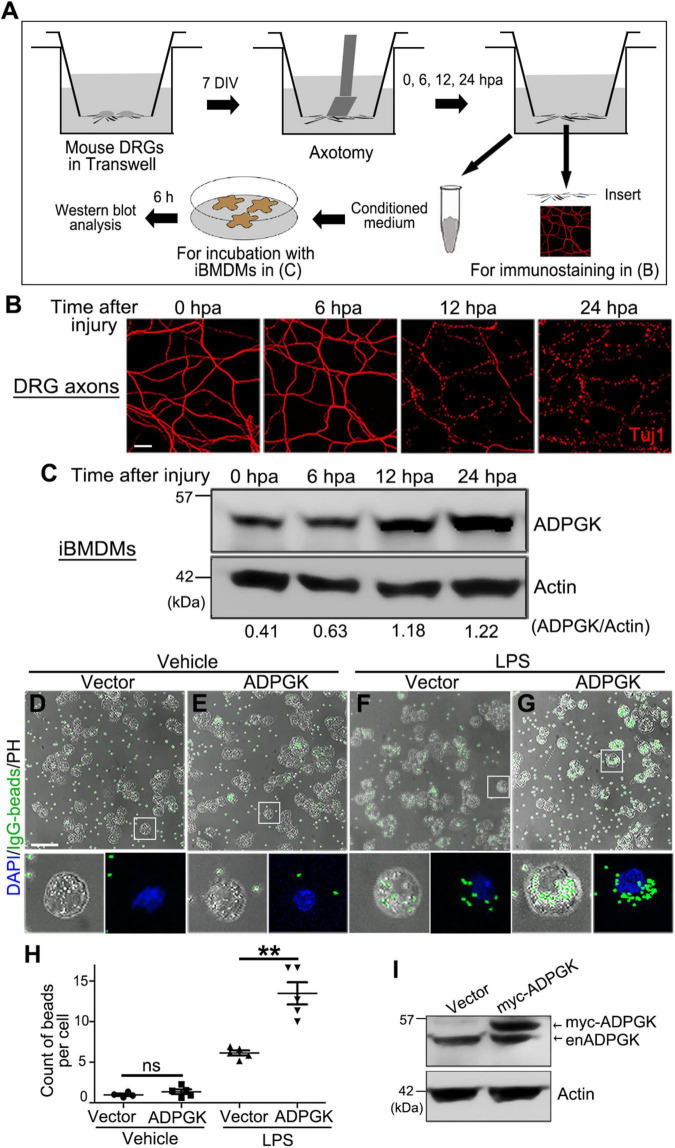
Injured axons induce ADPGK upregulation in macrophages non-cell autonomously and OE of ADPGK promotes phagocytosis. **(A)** A schematic diagram of deriving conditioned medium using the Transwell dish and an *in vitro* injury model of mouse DRG axons and treating the iBMDMs with the axon-derived medium. DIV, days *in vitro*. hpa, hours post axotomy. **(B)** Representative confocal images of mouse DRG axons at indicated time points after axotomy. Axonal integrity is examined by immunostaining for Tuj1. **(C)** The representative western blot analysis of ADPGK protein levels in the iBMDMs that are incubated with the conditioned media derived at indicated time points after axotomy. Shown is a representative image of three independent repeats. **(D–G)** The representative images of iBMDMs transfected with the empty vector (Vector) or *myc-ADPGK* (ADPGK) engulfing IgG-coated latex beads (green). The cells are treated with PBS (Vehicle) or LPS for 24 h. PH, phase contrast. **(H)** The average counts of beads per cell in **(D–G)** are quantified. **(I)** Western blot analysis confirming the lentiviral expression of *myc-ADPGK* in iBMDMs in **(D–G)**. enADPGK, endogenous ADPGK. Means ± SEM; *n* = ∼100 cells from pooled results of 5 repeats per group. Student’s *t*-test; **p < 0.01; ns, not significant. Scale bars: 25 μm in **(B)** and 50 μm in **(D–G)**.

To address the above question, we utilized the *in vitro* cultures of iBMDMs and performed the conditioned medium assay (Figure 4A; also see section “Materials and Methods”). In brief, mouse DRG explants were cultured *in vitro* in the Transwell dish for 7 days before the DRG somas in the upper compartment were removed. The integrity of injured DRG axons on the lower surface of the Transwell insert was examined at different time points by immunostaining. In this *in vitro* model, the severed DRG axons were mostly intact at 6 h post axotomy (hpa), significantly degenerated at 12 hpa and completely fragmented at 24 hpa (Figure 4B).

Meanwhile, we collected the axon-derived media in the lower compartment of the Transwell at 0, 6, 12, and 24 hpa and incubated the medium with iBMDMs for 6 h. We then examined the protein levels of ADPGK in these iBMDMs by western blotting. Compared to the iBMDMs incubated with the axon-derived medium at 0 hpa, the protein levels of ADPGK in the iBMDMs incubated with the conditioned media from injured axons were progressively increased over time, especially at 12 and 24 hpa (Figure 4C). The results indicated that degenerating axons could induce ADPGK upregulation in macrophages non-cell autonomously, likely by secreting some signaling molecules into the extracellular space.

### Increase of ADP-Dependent Glucokinase Levels Promotes Phagocytosis of Activated Macrophages

Macrophages are the effector cells of the innate immune system and the professional phagocytes highly specialized in removal of cellular debris and dead/dying cells (Kolliniati et al., 2021; Nazareth et al., 2021). To understand the functional relevance of ADPGK in macrophages in response to injury, we performed a macrophage phagocytosis assay using the engulfment of the IgG-coated beads as a readout (see section “Materials and Methods”). Interestingly, we found that overexpression (OE) of ADPGK *per se* was insufficient to activate the iBMDMs to engulf the IgG beads (Figures 4D,E). However, when pre-treating the iBMDMs with LPS to activate macrophages (Meng and Lowell, 1997; Fang et al., 2004), OE of ADPGK significantly promoted phagocytosis (Figures 4F–H). The expression of myc-ADPGK in iBMDMs by lentiviral infection was confirmed by western blotting (Figure 4I).

Together, with the quantitative proteomic analysis, we revealed a profound change of nerve proteins and potential participants in neural injury using a mouse SNI model in this study. By linking the results from both *in vivo* experiments and cell culture-based assays, we further confirmed that ADPGK was significantly increased in the sciatic nerve after injury and severed axons could induce ADPGK upregulation in macrophages non-cell autonomously. Finally, we showed that OE of ADPGK enhanced the phagocytotic activity of LPS-induced but not inactive macrophages, suggesting that upregulation of ADPGK might promote the engulfment of axon/myelin debris by macrophages in injury but was not involved in the initial activation of macrophages in response to the neural insult.

The current study may set an example for future mechanistic investigations on nerve injury by use of MS-based, quantitative proteomics. Meanwhile, the identified significantly changed proteins may serve as potential targets for developing novel treatments for PNI as well as other neurological disorders associated with peripheral nerve degeneration.

## Data Availability Statement

The mass spectrometry proteomics data have been deposited to the ProteomeXchange Consortium (http://proteomecentral.proteomexchange.org) via the iProX partner repository (Ma et al., 2019) with the dataset identifier PXD028726. All other data are included in the article and Supplementary Material.

## Ethics Statement

The animal study was reviewed and approved by the Interdisciplinary Research Center on Biology and Chemistry, the Chinese Academy of Sciences.

## Author Contributions

YZ, AL, and YF conceived the research. KZ, QW, YZ, and YF designed the experiments. KZ, QW, YL, YY, and HW performed the experiments. QW, XC, and AL contributed important new reagents. KZ, QW, YY, YZ, and YF analyzed the data and interpreted the results. KZ, QW, and YF prepared the figures. KZ, QW, YL, YY, YZ, AL, and YF wrote the manuscript. All authors read and approved the final manuscript.

## Conflict of Interest

The authors declare that the research was conducted in the absence of any commercial or financial relationships that could be construed as a potential conflict of interest.

## Publisher’s Note

All claims expressed in this article are solely those of the authors and do not necessarily represent those of their affiliated organizations, or those of the publisher, the editors and the reviewers. Any product that may be evaluated in this article, or claim that may be made by its manufacturer, is not guaranteed or endorsed by the publisher.

## Footnotes

1
https://david.ncifcrf.gov

2
http://www.geneontology.org

3
www.ensembl.org
