# Supplementary material for: Quantitative Proteomic Analysis of Mouse Sciatic Nerve Reveals Post-injury Upregulation of ADP-Dependent Glucokinase Promoting Macrophage Phagocytosis
Source: Front Mol Neurosci. 2021 Nov 12;14:777621. doi: 10.3389/fnmol.2021.777621 (PMC8633568; doi:10.3389/fnmol.2021.777621)
Supplement: Supplementary file 1 [file Image_1.pdf]

SUPPLEMENTARY INFORMATION

Quantitative proteomic analysis of mouse sciatic nerve reveals post-injury upregulation of ADP-dependent glucokinase promoting macrophage phagocytosis

Kai Zhang, Qingyao Wang, Yiyao Liang, Yu Yan, Haiqiong Wang, Xu Cao, Bing Shan, Yaoyang Zhang, Ang Li, and Yanshan Fang

Correspondence to: fangys@sioc.ac.cn

Supplementary Information includes:

Supplementary Figures S1 to S2 and the legends

Supplementary Tables S1 to S4 (in separate excel files)

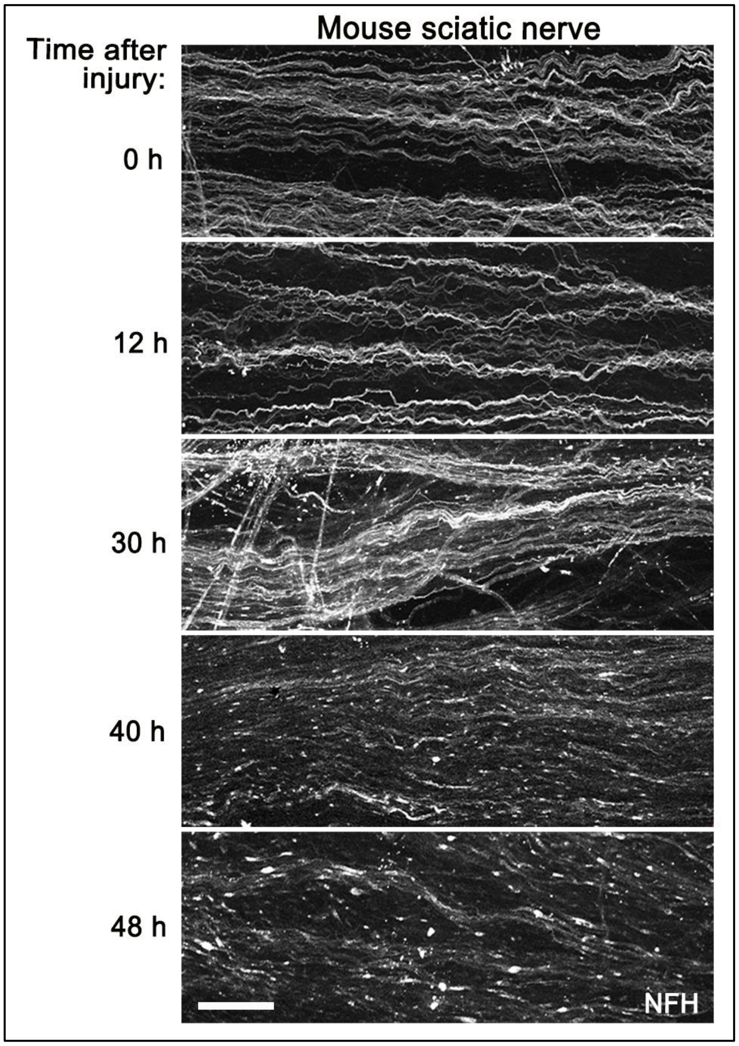

Figure S1. The time course of axonal degeneration of injured mouse sciatic nerve.

Whole-mount immunostaining on the mouse sciatic nerve at indicated time points after the crush injury. The axonal integrity is examined by immunostaining for neurofilament heavy chain (NFH), a protein marker for the neuro-axonal compartment. The mouse sciatic nerve starts to degenerate in between 30 and 40 hours after injury, evident by beading, disintegration and fragmentation of injured axons. Scale bar: 100 μm.

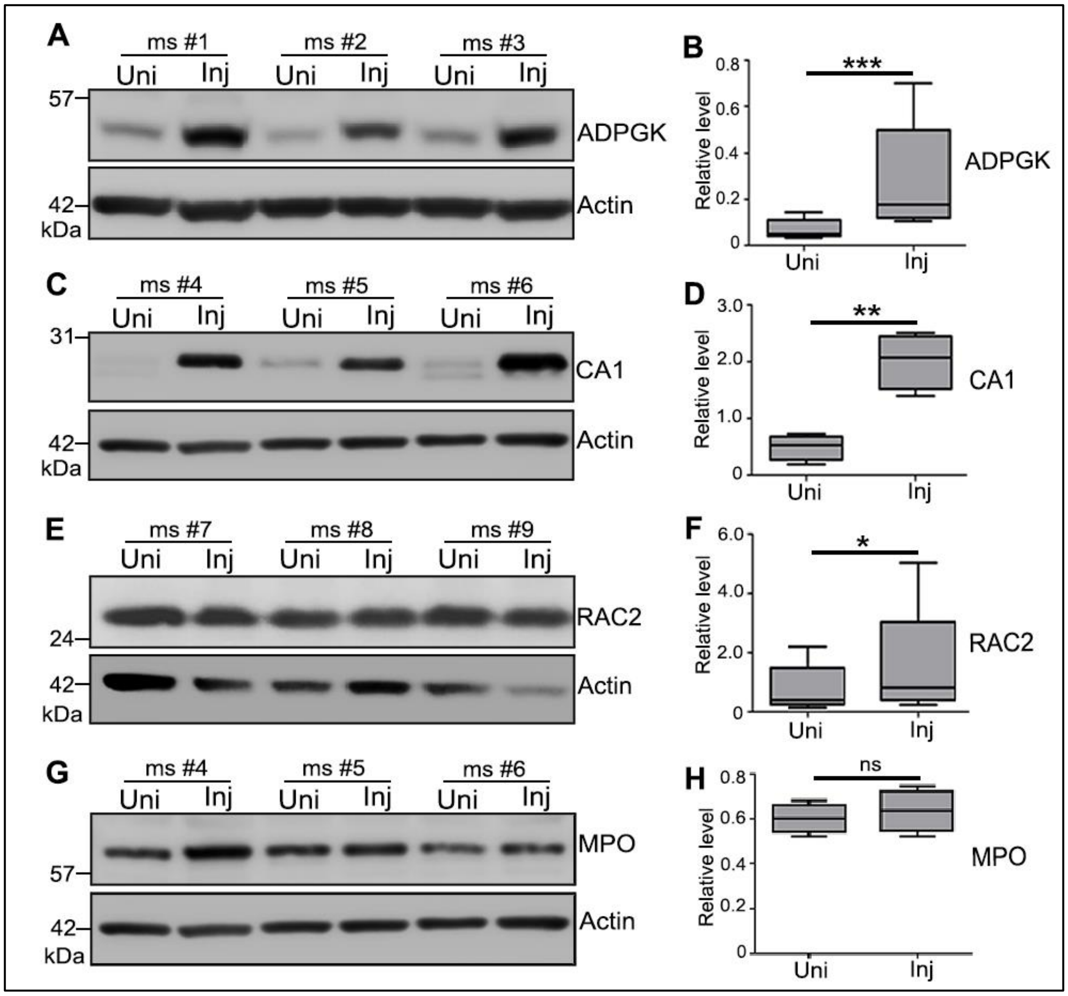

Figure S2. Validation of the proteomic analysis-identified, significantly changed proteins in the injured mouse sciatic nerve.

(**A-H**) The protein levels of four candidates in the list in Figure 2J were examined at 12 h after SNI by western blotting, including ADPGK (A-B), CA1 (C-D), RAC2 (E-F) and MPO (G-H). All protein levels are normalized to Actin. Uni, uninjured; Inj, injured. ms, mouse. At least 3 different mice were examined and the specific animal# tested in each assay is shown. Note that the same blot of Actin is shown in (C) and (G) because the protein samples from the same mice (#4 - #6) were separated in one SDS-PAGE gel and blotted with the antibodies for CA1 and MPO, respectively. Statistical significance is determined by paired *t*-test; $*p < 0.05$, $**p < 0.01$ and $***p < 0.001$; ns, not significant.
